# Mitochondrial Network Determines Intracellular ROS Dynamics and Sensitivity to Oxidative Stress through Switching Inter-Mitochondrial Messengers

**DOI:** 10.1371/journal.pone.0023211

**Published:** 2011-08-04

**Authors:** Junseong Park, Jungsul Lee, Chulhee Choi

**Affiliations:** 1 Department of Bio and Brain Engineering, KAIST, Daejeon, Korea; 2 Graduate School of Medical Science and Engineering, KAIST, Daejeon, Korea; 3 KAIST Institute for the BioCentury, KAIST, Daejeon, Korea; University of Windsor, Canada

## Abstract

Oxidative stresses caused by reactive oxygen species (ROS) can induce rapid depolarization of inner mitochondrial membrane potential and subsequent impairment of oxidative phosphorylation. Damaged mitochondria produce more ROS, especially the superoxide anion (O_2_^−^) and hydrogen peroxide (H_2_O_2_), which potentiate mitochondria-driven ROS propagation, so-called ROS-induced ROS release (RIRR), via activation of an inter-mitochondria signaling network. Therefore, loss of function in only a fraction of mitochondria might eventually affect cell viability through this positive feedback loop. Since ROS are very short-lived molecules in the biological milieu, mitochondrial network dynamics, such as density, number, and spatial distribution, can affect mitochondria-driven ROS propagation. To address this issue, we developed a mathematical model using an agent-based modeling approach, and tested the effect of mitochondrial network dynamics on RIRR for mitochondria under various conditions. Simulation results show that the intracellular ROS signaling pattern, such as ROS propagation speed and oxidative stress vulnerability, are critically affected by mitochondrial network dynamics. Mitochondrial network dynamics of mitochondrial distribution, density, activity, and size can mediate inter-mitochondrial signaling under certain conditions and determine the identity of the ROS signaling pattern. We further elucidated the potential mechanism of these actions, i.e., conversion of major messenger molecules involved in ROS signaling. If the average distance between neighboring mitochondria is large or mitochondrial distribution becomes randomized, messenger molecule of the ROS signaling network can be switched from O_2_^−^ to H_2_O_2_. In this case, mitochondria-driven ROS propagation is efficiently blocked by introduction of excess cytosolic glutathione peroxidase 1, while introduction of cytosolic superoxide dismutase has no effect. Together, these results suggest that mitochondrial network dynamics is a major determinant for cellular responses to RIRR through changing the key messenger molecules.

## Introduction

Among many intracellular organelles, mitochondria function mainly in production of ATP through oxidative phosphorylation in the inner mitochondrial membrane. During this respiration process, reactive oxygen species (ROS) are also produced when high-energy electrons escape before they reach the final acceptor, O_2_ [1]. Even under normal conditions, 1-4% of oxygen is reduced in mitochondria by a one-electron reduction to generate ROS, specifically superoxide anion (O_2_^−^), which can further mediate oxidative damage to many intracellular components including proteins, nucleic acids, and lipid membranes [2], [3]. To protect macromolecules from oxidative modification, cells have developed antioxidant defense systems. The first ROS produced in mitochondria is the highly reactive O_2_^−^, and superoxide dismutase (SOD) converts this into a much more stable and therefore relatively inert ROS, hydrogen peroxide (H_2_O_2_). H_2_O_2_ is reduced to water (H_2_O) by many antioxidant enzymes such as catalase, peroxiredoxin (Prx), and glutathione peroxidase (Gpx) [2], [4], [5].

**Figure 1:**
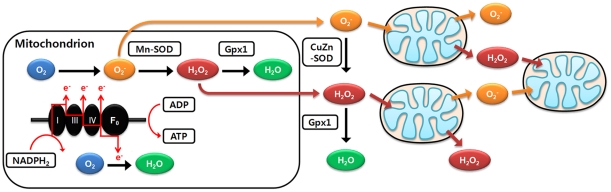
Schematic diagram of RIRR and mitochondria-driven ROS propagation. Oxidative phosphorylation occurring in the mitochondrial electron transport chain is an ATP producing mechanism. During this respiration process, ROS are also produced when high-energy electrons escape before they reach the final acceptor O_2_. ROS induce a rapid depolarization of mitochondrial inner membrane potential and subsequent impairment of oxidative phosphorylation. Damaged mitochondria produce more ROS, especially the superoxide anion (O_2_^−^) and hydrogen peroxide (H_2_O_2_), which potentiates RIRR and mitochondria-driven ROS propagation via activation of an inter-mitochondria signaling network. Therefore, loss of function in only a fraction of the mitochondria may eventually affect the viability of the whole cell through this positive feedback loop.

Production of ROS in mitochondria is accelerated by ROS themselves. This phenomenon is called ROS-induced ROS release (RIRR), and ROS generation in only small numbers of mitochondria can affect neighboring mitochondria, eventually propagating the ROS surge to the whole cell through this positive feedback loop (Figure 1) [6], [7]. In cardiomyocytes, intracellular ROS propagation involves strongly reactive O_2_^−^ as the main messenger molecule [6], [8], [9]. For neurons, however, conflicting studies have reported that overexpression of SOD, which is a specific antioxidant for O_2_^−^, could exacerbate oxidative stress-mediated neuronal cell death [10], [11]. Our experimental results show that treatment of cells with a SOD mimetic, MnTBAP, under oxidative stress triggers abnormal hyperpolarization of mitochondria (unpublished data). Also, reduction of CuZn-SOD is known to lower H_2_O_2_ toxicity, resulting in increased resistance to oxidative stress in GMF-null astrocytes [12]. These reports contradict the general understanding that the inter-mitochondrial messenger molecule for RIRR is O_2_^−^. We hypothesized that under certain conditions, H_2_O_2_ might be a more appropriate messenger molecule to propagate ROS and cause whole-cell RIRR because of its longer lifetime in the cytosol and higher permeability in membrane lipids [13], [14]. Based on the reports that mitochondria form a dynamic network responsible for energy production, calcium homeostasis and cell signaling [15], we devised an inter-mitochondrial network model using agent-based modeling (ABM) to examine this hypothesis.

ABM is a mathematical and computational technique that simulates interactions between agents within a complex system. It has a rule-based modeling format that determines probabilities to next states at every step, unlike ordinary differential equation (ODE) modeling. Each agent in ABM has several attributes and a set of predefined reaction rules, and therefore executes a series of operations. ABM takes relatively longer simulation time than ODE, but is advantageous to implement in biological systems (e.g., for time delay reactions, spatial translocation, and biased distribution) as it does not require conversion to an equation [16], [17]. Because our model includes variables that have spatial information, such as the location of each mitochondrion and molecule, as well as temporal information, the ABM method is appropriate for our subject. In the following sections, we provide detailed simulation results and describe use of the ABM in mitochondrial network dynamics and ROS propagation.

## Results

### Different responses to oxidative stress by mitochondrial network dynamics

**Figure 2:**
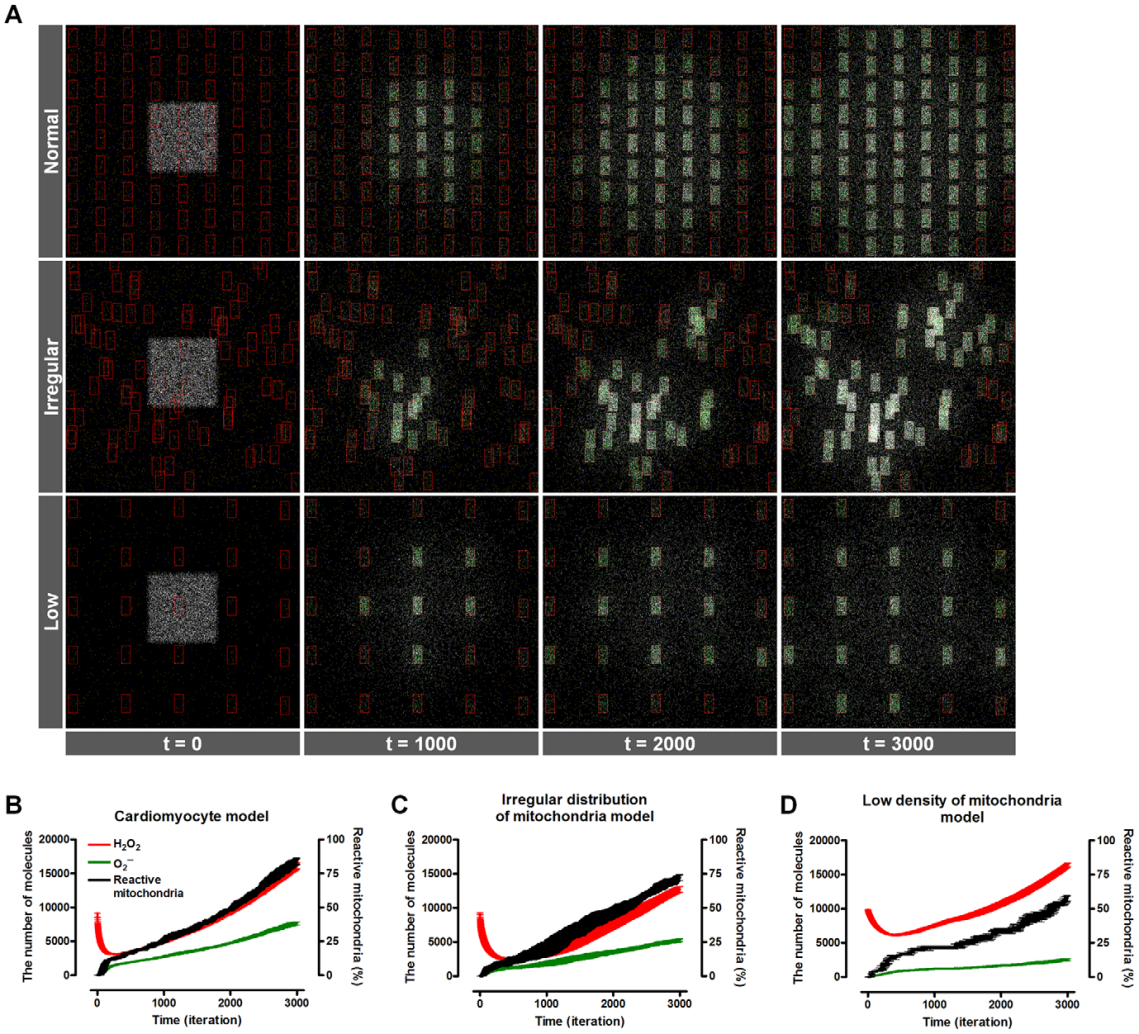
Simulation of the mitochondrial network model implemented using an ABM method. (A) Captured images of real simulation steps in the cardiomyocyte model (top panel), the irregular distribution of mitochondria model (middle panel), and the low density of mitochondria model (bottom panel). Red squares indicate a mitochondrion, white particles represent H_2_O_2_, and green particles denote O_2_^−^. The model also includes intermediate molecules and three types of antioxidant enzyme, SOD, Gpx1, and catalase. (B–D) Graph shows time course behavior of a model containing five simulation results.

Given oxidative stress, ROS act as messenger molecules and further affect neighboring mitochondria to produce more ROS, which eventually propagate throughout the whole cell (Figure 1). In this context, mitochondrial network dynamics provides critical determinants for cellular response to oxidative stress. Our results show that the speed of ROS propagation in the cytosol, dose dependency to initial oxidative stress, and overall sensitivity to oxidative stress change in relation to mitochondrial network dynamics. We investigated how these characteristics were affected by modifying two major variables of mitochondrial network dynamics: the mitochondrial distribution pattern in the cytosol and the number of mitochondria within the cell. Regular distribution of mitochondria is defined as a uniform distance between mitochondria, as reported for cardiomyocytes (Figure 2A, top), whereas an irregular distribution of mitochondria implies a random distribution of mitochondria in the cytosol (Figure 2A, middle). Because mitochondria are irregularly scattered in neurons, for example, this simulation implies a real-world comparison between cardiomyocytes and many other cell types that have irregular distribution of mitochondria.

The number of mitochondria within a cell differs substantially according to the cell type, and depends on how much energy the cell needs to produce. Cells with particularly heavy energy demands (such as cardiomyocytes) contain more mitochondria compared to other cell types. To examine how the cytosolic density of mitochondria influences the cell's response to oxidative stress, the degree of ROS propagation was measured for a cardiomyocyte model and for a reduced number (81 to 25 per cell) of mitochondria (Figure 2A, bottom). We compared these three models of mitochondrial network dynamics: a regular distribution, an irregular distribution, and a low cytosolic density of mitochondria.

At the start of the simulation, H_2_O_2_ was introduced into a restricted center area of the cytosol to mimic initial oxidative stress. This oxidative stress affects mitochondria in that local region, and affected mitochondria produce more ROS that propagate and affect neighboring mitochondria, eventually increasing the total intracellular ROS. As the simulation proceeds, ROS propagation either causes whole-cell RIRR or is efficiently blocked by antioxidant enzyme systems, depending on the amount of initial oxidative stress and mitochondrial network dynamics.

**Figure 3:**
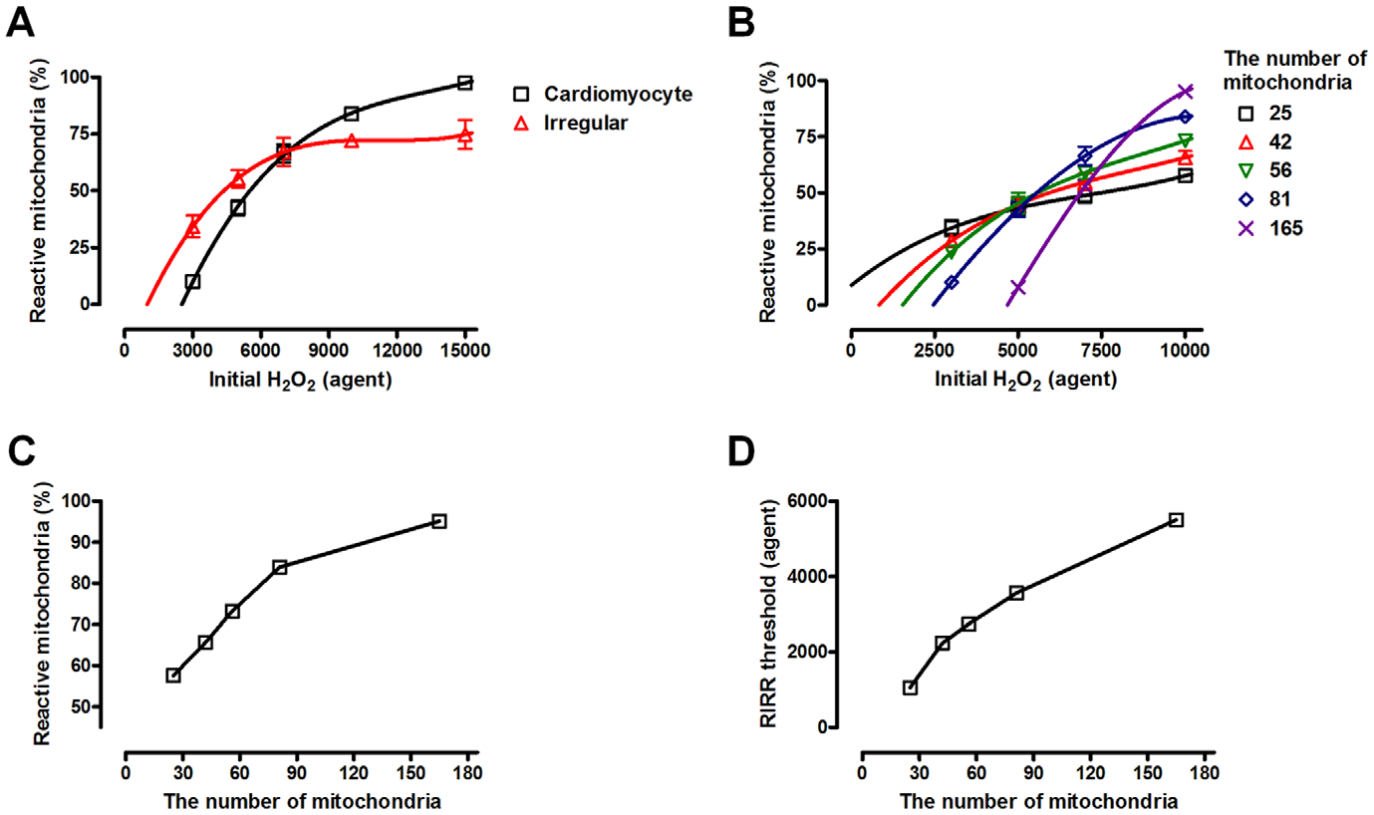
Dose dependence on the oxidative stress. (A) At a time point 3000 (the number of simulation steps), the cardiomyocyte model shows a steeper gradient than the irregular distribution of mitochondria model. (B) Dose-dependence on the initial oxidative stress is also tested for different cytosolic densities of mitochondria (regular distribution). The higher cytosolic number of mitochondria model is more sensitive to initial oxidative stress. The third order polynomial equations are used for curve fitting in (A) and (B) (C) When initial H_2_O_2_ is sufficiently high (10000), the fraction of reactive mitochondria increases according to the number of mitochondria, indicating a significant dose-dependency. (D) RIRR threshold is defined by the initial H_2_O_2_ of the regression estimate for reactive mitochondria  =  20%.

However, the dose dependency on initial oxidative stress differs according to the type of mitochondrial network dynamics (Figure 3). When initial oxidative stress is strong, the increase in reactive mitochondria and mitochondria-driven ROS propagation is more rapid in the cardiomyocyte model than in the irregular distribution model. At time point 3000 (the number of simulation steps), more fractions of mitochondria are reactive and affected by ROS in the cardiomyocyte model, indicating that ROS propagation is faster compared to the irregular distribution model. However, given low initial H_2_O_2_ levels, a cardiomyocyte model is more resistant to ROS propagation, whereas an irregular distribution model is prone to trigger propagation (Figure 3A). In the irregular model, high mitochondrial density region is susceptible to oxidative stress and ready to propagate ROS, thus low level of initial oxidative stress is sufficient to induce whole-cell RIRR. However, low mitochondrial density region is relatively isolated from inter-mitochondrial network, making resistance to high oxidative stress. Therefore, the cardiomyocyte model has higher dose dependency regarding the initial oxidative stress compared to the irregular distribution model.

Dose-dependency on the initial oxidative stress was also tested with different cytosolic densities of mitochondria in the regular distribution (Figure 3B). A low density of mitochondria makes cells vulnerable to ROS propagation at mild initial oxidative stress compared to cells with a high density of mitochondria. However, an additional high dose of initial oxidative stress does not cause a significant increase in reactive mitochondria and mitochondria-driven ROS propagation. Rather, cells with a high density of mitochondria shows rapid ROS propagation and whole-cell RIRR after strong initial oxidative stress, indicating a positive correlation between the number of mitochondria and dose dependency on the initial oxidative stress. As a result of this difference in dose dependency, the initial H_2_O_2_ threshold that can trigger RIRR (RIRR threshold) and whole-cell ROS propagation is higher in the high density of mitochondria model than in the low density of mitochondria model (Figure 3C and D). The RIRR threshold at specific mitochondria density is defined by the initial H_2_O_2_ of the regression estimate at reactive mitochondria  = 20% (The third order polynomial equation is used for the nonlinear regression as described in *Material and Methods*). Hence, the same input of oxidative stress can cause different responses, and the eventual fate of cells is related to mitochondrial network dynamics.

### Conversion of main messenger ROS molecules through mitochondrial network dynamics

**Figure 4:**
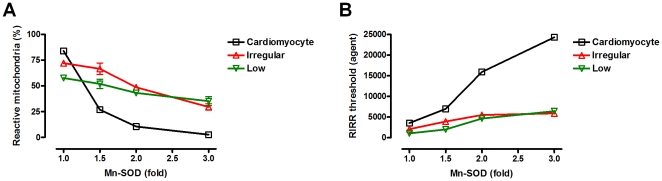
Effects of increased Mn-SOD in each type of mitochondrial network dynamics. (A) Fractions of reactive mitochondria according to the amount of Mn-SOD are shown at time point 3000 (the number of simulation steps). The x-axis indicates the amount of Mn-SOD, and the y-axis shows the fractions of reactive mitochondria involved in RIRR and mitochondria-driven ROS propagation. ROS propagation is more efficiently blocked by higher doses of Mn-SOD in the cardiomyocyte model than in the irregular distribution and low number of mitochondria models. (B) RIRR thresholds according to the amount of Mn-SOD are shown for each type of mitochondrial network dynamics. RIRR threshold values are built up more rapidly by increased amounts of Mn-SOD in the cardiomyocyte model than in the irregular distribution and low number of mitochondria models. The second order polynomial equations are used for nonlinear regression and calculation of RIRR threshold values. The x-axis indicates the amount of Mn-SOD, and the y-axis shows RIRR threshold values.

We hypothesized that results in Figure 3 are caused by conversion of the main messenger ROS molecules working on ROS signaling. To confirm this, we modulated various antioxidant systems in the cell: mitochondrial Mn-SOD, cytosolic CuZn-SOD, and Gpx1 (only cytosolic Gpx1). Because Mn-SOD converts O_2_^−^ to the much less reactive H_2_O_2_ in mitochondria, it can protect cells from oxidative stress. However, if ROS propagation occurs in a H_2_O_2_-dependent manner, Mn-SOD will not provide significant protection or may even exacerbate overall stress. Figure 4 shows the effect of Mn-SOD for each type of mitochondrial network dynamics. In the cardiomyocyte model, raised expression levels of Mn-SOD remarkably reduce reactive mitochondria, and further ROS propagation (Figure 4A). Also, the RIRR threshold values (defined as in Figure 3: i.e., initial H_2_O_2_ of the nonlinear regression estimate at reactive mitochondria  = 20% by the second order polynomial equations) are built up rapidly by increased amounts of Mn-SOD (Figure 4B). However, these protective effects of Mn-SOD are largely abolished in the irregular distribution and low density of mitochondria models. This implies that changes in mitochondrial network dynamics cause a transition of the ROS signaling pattern through conversion of the main messenger ROS molecules. We consider that the main messenger ROS molecules are O_2_^−^ in the cardiomyocyte, whereas they switch to H_2_O_2_ in the irregular distribution and low number of mitochondria cases. Moreover, the data provide a plausible mechanism of SOD cytotoxicity in certain types of cells and in disease models [10], [11].

**Figure 5:**
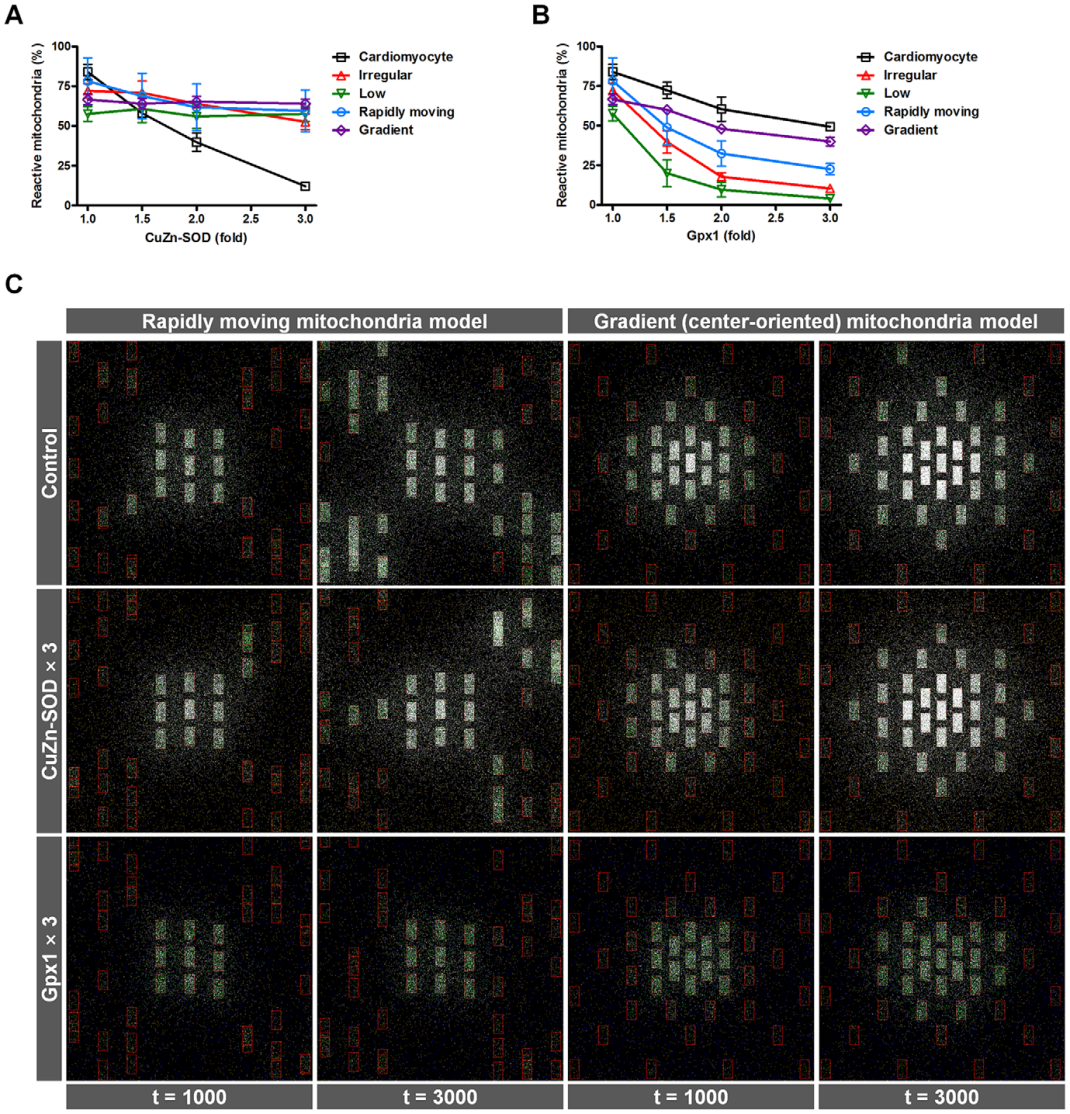
Change of ROS propagation and messenger molecules by mitochondrial distribution and cytosolic density of mitochondria. (A–B) Fractions of reactive mitochondria according to the amount of CuZn-SOD (A) and cytosolic Gpx (B) are shown at a specific time point (the number of simulation steps  = 3000). The x-axis indicates the amount of each cytosolic antioxidant, and the y-axis indicates the fractions of reactive mitochondria involved in RIRR and mitochondria-driven ROS propagation. (C) Captured images of real simulation steps in the rapidly moving and gradient mitochondria models with control (top panel), increase in CuZn-SOD (middle panel), or cytosolic Gpx (bottom panel). In the rapidly moving mitochondria model, peripheral mitochondria were defined to move vertically at each simulation step.

We further confirmed the hypothesis that the main messenger ROS molecules switch from O_2_^−^ to H_2_O_2_ in the irregular distribution and low number of mitochondria models. We also added irregularly, but not randomly distributed mitochondria models. Rapidly moving mitochondria model mimics neuron, whose mitochondrial movement is known to be highly dynamic and very important to maintain local ATP levels at the synapse [15], [18]. Gradient (center-oriented) mitochondria model implies mitochondrial clustering surrounding the nucleus for an integrated phospho-transfer network and energetic channeling between mitochondria and nuclei [19]. For each simulation, the amount of the cytosolic antioxidants, CuZn-SOD and Gpx1 (only cytosolic Gpx1) was modulated, and ROS propagation was measured. This method allows determining the main ROS molecules working on ROS propagation and whole-cell RIRR. If ROS propagation is blocked when the amount of CuZn-SOD is raised, it is an O_2_^−^ dependent network because CuZn-SOD is an antioxidant enzyme that converts O_2_^−^ to H_2_O_2_ in the cytosol. Similarly, if an increase in cytosolic Gpx1 can block ROS propagation, then H_2_O_2_-dependent RIRR occurs because Gpx1 is an antioxidant that converts H_2_O_2_ to water. In a simulation, an increase in CuZn-SOD can block ROS propagation in the cardiomyocyte model. However, in the irregular distribution, low density, rapidly moving, and gradient mitochondria models, ROS propagation is not blocked by an increase in CuZn-SOD (Figure 5A). In contrast to CuZn-SOD, Gpx1 has a significant cyto-protective effect in the irregular distribution, low density mitochondria, and rapidly moving mitochondria models, whereas the effect in the cardiomyocyte model is subtle (Figure 5B). Even though an increase in Gpx1 is not effective to block ROS propagation, concurrent increase in SOD and Gpx efficiently inhibits mitochondria-driven ROS propagation (data not shown). Captured images of the real simulation clearly show how each antioxidant prevents ROS propagation (Figure 5C, only the rapidly moving and gradient mitochondria models are shown). Together, the data show a correlation between mitochondrial network dynamics and main messenger ROS molecules. When mitochondrial distribution is regular and cytosolic density of mitochondria is high (cardiomyocyte model), the main messenger ROS molecules are O_2_^−^, consistent with previous research on cardiomyocytes [9], [20]. However, the effect of each antioxidant is reversed when the mitochondrial distribution is irregular or cytosolic density is low, implying that the main messenger ROS molecule working on whole-cell RIRR changes from O_2_^−^ to H_2_O_2_, thus, RIRR is H_2_O_2_-dependent. As expected from these simulation results, when mitochondrial distribution is irregular and the cytosolic density of mitochondria is low simultaneously, the main messenger ROS molecules are H_2_O_2_ (data not shown). Therefore, we conclude that mitochondrial network dynamics including mitochondrial distribution and cytosolic density of mitochondria are critical determinants of inter-mitochondrial ROS signaling patterns and main messenger ROS molecules.

### Different kinetics of H_2_O_2_ and O_2_^−^

**Figure 6:**
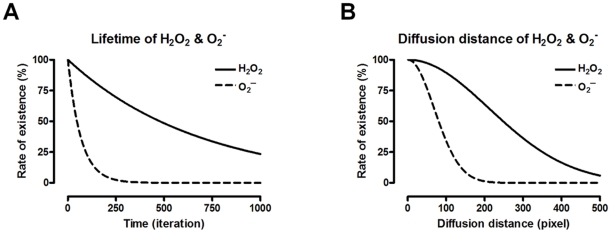
Different kinetics of H_2_O_2_ and O_2_^−^. The lifetime of each type of ROS molecule is calculated from the many parameters employed in the model (A). Graph shows that H_2_O_2_ has a much longer life than O_2_^−^. This result is used to estimate how far each ROS molecule can travel in the cytosol (B). The result shows that H_2_O_2_ has a longer average diffusion distance and higher probability to reach neighboring mitochondria than O_2_^−^.

The reason why the main messenger ROS molecules are altered by the conditions affecting mitochondrial network dynamics is the different kinetics of the two ROS types, O_2_^−^ and H_2_O_2_. From antioxidant-dependent, spontaneous degradation rate, and many other parameters used in the model, we calculated decay rate of each ROS molecule in a single simulation step. With this value, the lifetime of each ROS molecule in the cytosol is calculated (Figure 6A). This allows estimation of the average distance that each ROS molecule can travel in the cytosol (Figure 6B). Mean-square diffusion displacement of particles moving with a random walk algorithm in two-dimensional (2D) space is induced as <(x_N_)^2^>  = 2NL^2^. Here, x_N_ is the displacement of each agent for N time steps, and L is the maximum displacement for one time step (see Materials and Methods for details). As shown in Figure 6, O_2_^−^ has a shorter lifetime than H_2_O_2_, and therefore, H_2_O_2_ can travel much farther in the cytosol. These characteristics give H_2_O_2_ an advantage in ROS signaling compared to O_2_^−^ when the distance between mitochondria is large.

**Figure 7:**
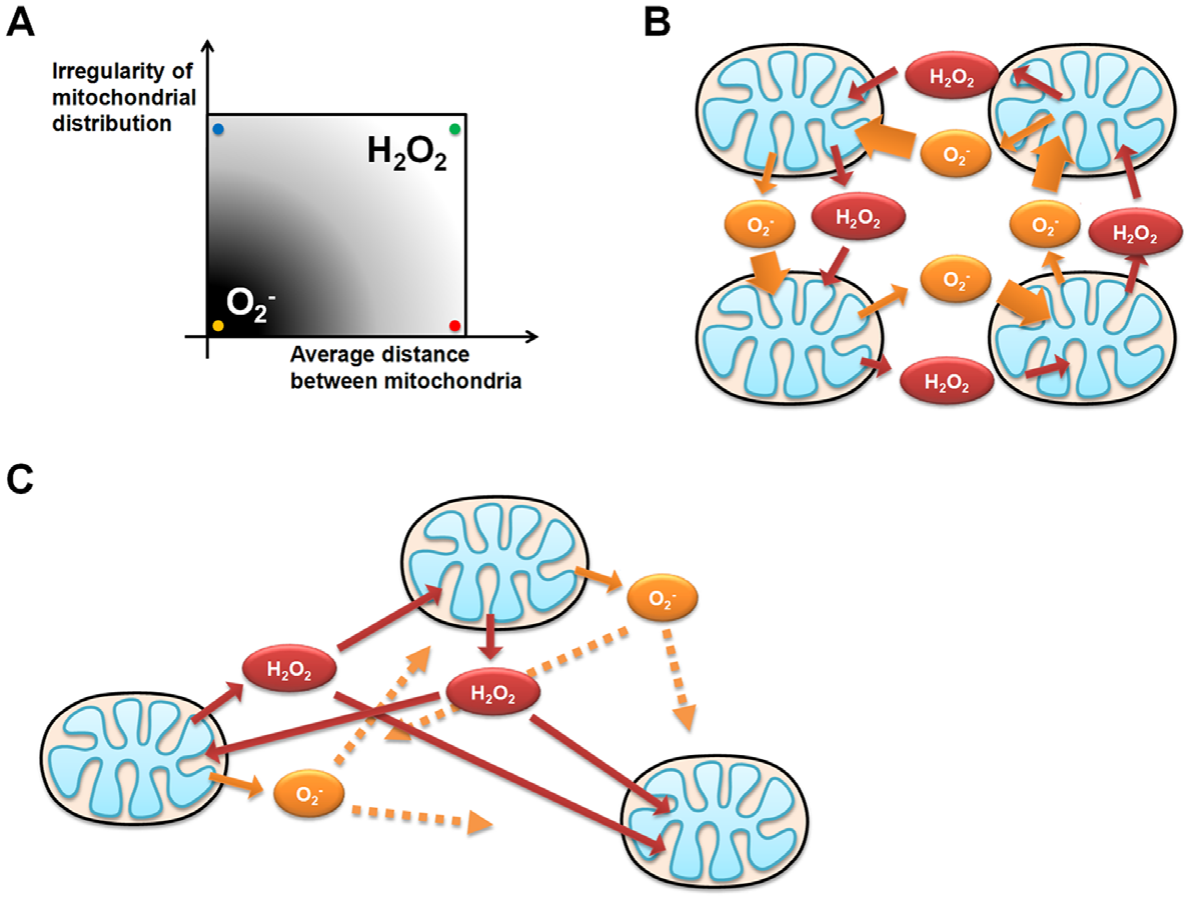
Main messenger ROS molecules acting on mitochondria-driven ROS propagation. (A) Previous simulation results are summarized for the effect of two macro-variables, which are mitochondrial distribution and the number of mitochondria, on inter-mitochondrial ROS messenger molecules (mitochondrial distribution is dichotomous variable, so tested for regular and irregular cases). When mitochondrial distribution is regular and the number of mitochondria is relatively high (cardiomyocyte model, yellow spot), inter-mitochondrial ROS propagation is O_2_^−^ dependent. However, the main messenger ROS molecules are converted to H_2_O_2_ when mitochondrial distribution is irregular (blue spot) or the cytosolic density of mitochondria becomes relatively low (red spot). (B) Mitochondria-driven ROS propagation pattern of the yellow spot case in (A) (C) RIRR pattern of the green spot case in (A).

Finally, schematic diagram of main messenger ROS molecules working on mitochondria-driven ROS propagation well summarizes this research. We demonstrated cardiomyocyte model (yellow spot case in Figure 7A), irregular distribution of mitochondria model (blue spot case in Figure 7A), and low density of mitochondria model (red spot case in Figure 7A). Because of different kinetics of O_2_^−^ and H_2_O_2_, main ROS molecules working on RIRR are changed by inter-miochondrial network dynamics (Figure 7B and C).

## Discussion

We developed a mathematical model describing the inter-mitochondrial network, considering the location of each mitochondrion and propagation of ROS molecules using ABM. Simulation results clearly showed that mitochondrial network dynamics could determine intracellular ROS propagation profiles and cellular sensitivity to oxidative stress by converting the main messenger ROS molecules acting on inter-mitochondrial ROS communication.

Several studies have reported on RIRR and how loss of function in a small number of mitochondria might influence overall cell functioning [6], [7], [21]. Especially, L.Zhou et al. developed mathematical model of RIRR based on reaction-diffusion and identified novel aspects of the inter-mitochondrial synchronization mechanisms [22]. However, most of previous RIRR studies were based on very specialized mitochondrial case implying cardiomyocytes, and many unknowns still exist regarding mitochondrial dynamics and mitochondria-driven ROS propagation. Moreover, some current experimental results do not fit the existing model. To resolve this problem, more systemic approaches to inter-mitochondrial networks are needed. The simulation results provided here clearly showed a good correlation between mitochondrial network dynamics and ROS propagation via a mechanism in which main messenger ROS molecules working on RIRR can be switched from O_2_^−^ to H_2_O_2_ under certain conditions. In the previous research, more reactive ROS molecules, especially O_2_^−^, were considered harmful and important messengers in the process of RIRR. Because such research on RIRR was performed using cardiomyocytes, which have a strictly regular and high number of mitochondria, this explanation is reasonable only in that specific system [6], [8], [9], [21], [22]. However, our simulation results revealed that mitochondrial network dynamics determine the identity of inter-mitochondrial ROS signaling and that H_2_O_2_ dependent ROS propagation occurs according to the distribution of mitochondria and distance between neighboring mitochondria. In this mechanism, ROS propagation speed, effect of different antioxidant enzymes, and vulnerability to oxidative stress deciding the ultimate fate of the cell can be determined according to mitochondrial network dynamics.

Our data can explain several extraordinary findings in some types of cell in which inhibition or reduction of CuZn-SOD rather increases resistance to oxidative stress [12], and treatment cells with the SOD mimetic MnTBAP triggers abnormal hyperpolarization of mitochondria under oxidative stress (unpublished data). Our data also provide a plausible explanation for the correlation between oxidative stress vulnerability and the status of the cytoskeleton in cardiomyocytes. Colchicine is reported to disrupt microtubules by binding to tubulin subunits [23]. Because mitochondria are attached to and move along these microtubules, colchicine may break up regular distribution of mitochondria. Therefore, our results also provide an answer to how colchicine attenuates ischemic preconditioning induced cardioprotection [24].

The ABM method makes it possible to control many variables of spatial information in molecular signaling systems. A Mathematical and computational approach based on ABM is meaningful and reasonable in that it provides many simulation results in diverse cases by independently controlling the variables, whereas design of *in vitro*/*in vivo* experiments in this subject would be very difficult. In practice, ABM has been applied in several fields, such as the social sciences, economics, and life sciences, especially oncology. To the best of our knowledge, we have pioneered the use of ABM for the first time in simulating inter-mitochondrial network dynamics and ROS signaling.

Based on the results present here, we propose that the main messenger ROS molecules acting on the propagation of ROS in the inter-mitochondrial network can be affected by the irregularity of mitochondrial distribution and average distance between mitochondria (Figure 7). In this mechanism, the degree of ROS propagation, effect of each antioxidant enzyme, and vulnerability to oxidative stress deciding the ultimate fate of a cell can be changed dependent on the characteristics of mitochondrial network dynamics. By introducing an ABM-based computational analysis, the complex dynamics of various cellular behaviors other than RIRR can also be better understood and might provide a rational background for developing of mechanism-based therapeutic interventions.

## Materials and Methods

### Modeling

Our model includes mitochondria, ROS, and antioxidant enzymes as agents, and exact amount of these agents are initially located in 2D space. Simulation proceeds with these agents that diffuse through a random walk algorithm in 2D space and independently react according to predefined rules. The concentrations of species are converted to percent (%) by the total number of each kind of species over the number of whole pixels in 2D space. Intra-mitochondria and cytosol are programmed to be different cellular compartments. When the ROS molecules meet this boundary, they can pass through it by predefined probabilities. The mitochondrion, which has more than 50 O_2_^−^ agents, is defined as the reactive mitochondrion.

Because this model employs stochastic simulation implying intrinsic randomness, each data set was obtained from five independent simulations, and the mean values and error bars were calculated. The endpoints of all simulations were 3000 (time steps). The model was developed in the Java language and simulated in a J2SE 5.0 environment.

### Parameter estimation

Cardiomyocytes have strictly regular and high numbers of mitochondria, and are known to be prone to RIRR [7], [21]. Parameters used in the model are extracted from many experimental results on cardiomyocytes, the PubChem database, and BRENDA database, and are described in Table 1. The activities of SOD and Gpx (rate constants) are used to determine the reaction speed when the substrates and the enzyme meet in the same pixel.

**Table 1:** Parameters used in model.

Biological parameters	Value	Reference/approximation
Maximum speed of H_2_O_2_	11.31 pixels per time step	calculated from PubChem
Maximum speed of O_2_^−^	11.66 pixels per time step	calculated from PubChem
Maximum speed of antioxidant enzymes	1.414 pixels per time step	calculated from PubChem
Spontaneous degradation of H_2_O_2_	0.001% per time step	calculated from [14], [28]
Spontaneous degradation of O_2_^−^	0.769% per time step	calculated from [14]
Spontaneous degradation of antioxidant enzymes	0 (neglectable)	
Respiration frequency per time step	0.01×size of an mitochondrion	suitably modulated
Electron leaking during normal respiration	1% per time step	[1], [14]
Maximum electron leaking in oxidative condition	20% per time step	estimated from [25], [26]
Electron leaking(O_2_^−^ generation) rule	0.01 + 0.19/(1 + exp(-concentration[H_2_O_2_]×0.01 - concentration[O_2_^−^]×0.09))	estimated from [14], [26], [27]
Concentration of Mn-SOD in mitochondria	0.769% (3×10^−6^M)	[5], [14]
Concentration of CuZn-SOD in cytosol	0.914% (3.56×10^−6^M)	[5], [14], [29]
Activity of SOD	2.3×10^9^/Ms	[14]
Concentration of Gpx in mitochondria	0.3% (1.17×10^−6^M)	[14]
Concentration of Gpx in cytosol	0.549% (2.14×10^−6^M)	[14], [29], [30]
Activity of Gpx	5×10^7^/Ms	[14]
Longitudinal distance between two mitochondria	18 pixels (0.4 µm)	[21]
Horizontal distance between two mitochondria	54 pixels (1.2 µm)	estimated from [21]
Longitudinal length of a mitochondrion	54 pixels (1.2 µm)	[21]

Parameters used in inter-mitochondrial network model are described.

### Electron leakage (O_2_^−^ generation) rule

The mitochondrial electron transfer chain has a basal level O_2_^−^ generation rate about 1∼4% [2], [3], and can reach up to 20% if mitochondria are damaged [25], [26]. In particular, ROS themselves accelerate O_2_^−^ generation through the RIRR mechanism, and different effects of each type of ROS molecule [14], [26], [27] are applied to the model. The electron leakage (O_2_^−^ generation) rule for each mitochondrion obeys a sigmoid function based on y ∝ 1/(1 + e^−x^), (‘x’ means ROS concentration, and ‘y’ indicates the probability of electron leakage) that has values from 1% to 20% depending on the number of ROS that mitochondrion contains.

### Diffusion law in random walks

We fixed a set of maximum diffusion speeds for all agents used in our model, and random values (-0.5 to 0.5) are multiplied in every cycle. Each molecule diffuses stochastically with this random walk method, and the mean-square diffusion displacement in 2D space can be calculated. If we suppose one-dimensional random walks and each step has a length L, the displacement of step N is k_N_L, where k_N_ is equally likely to be ±1. If the initial position is x_0_ = 0, then x_1_  =  k_1_L, and similarly the position after N steps is x_N_  =  x_N-1_ + k_N_L. Because each walk is random, the average of x_N_ (mean displacement of random walks) over many different trials is zero. However, the mean-square displacement is written as <(x_N_)^2^>  =  <(x_N-1_ + k_N_L)^2^>  =  <(x_N-1_)^2^> + 2L<x_N-1_k_N_> + L^2^<(k_N_)^2^>. Therefore, a walk of N steps has mean-square displacement larger by L^2^ than a walk of N-1 steps, which in turn is L^2^ larger than a walk of N-2 steps, and so on. Carrying this logic to its conclusion, we find that <(x_N_)^2^>  =  NL^2^. In 2D random walks, each step is a diagonal and has a length L√2. Hence, the mean-square diffusion displacement in 2D space is induced as <(x_N_)^2^>  = 2NL^2^.

### Nonlinear regression for curve fitting

To calculate the RIRR threshold values, a nonlinear regression is used for curve fitting. Among many functions, second order (y  =  A + Bx + Cx^2^) and third order (y  =  A + Bx + Cx^2^ + Dx^3^) polynomial equations give the best curve fitting results.
